# Large-Scale In Vitro Expansion of Polyclonal Human Switched-Memory B Lymphocytes

**DOI:** 10.1371/journal.pone.0051946

**Published:** 2012-12-17

**Authors:** Sonia Néron, Annie Roy, Nellie Dumont

**Affiliations:** 1 Héma-Québec, Ingénierie Cellulaire, Recherche et Développement, Québec, Québec, Canada; 2 Université Laval, Faculté des Sciences et de Génie, Département de Biochimie et Microbiologie, Québec, Québec, Canada; Institut National de la Santé et de la Recherche Médicale U 872, France

## Abstract

Polyclonal preparations of therapeutic immunoglobulins, namely intravenous immunoglobulins (IVIg), are essential in the treatment of immunodeficiency and are increasingly used for the treatment of autoimmune and inflammatory diseases. Currently, patients’ accessibility to IVIg depends exclusively upon volunteer blood donations followed by the fractionation of pooled human plasma obtained from thousands of individuals. Presently, there are no *in vitro* cell culture procedures allowing the preparation of polyclonal human antibodies. All *in vitro* human therapeutic antibodies that are currently generated are based on monoclonal antibodies, which are mostly issued from genetic engineering or single cell antibody technologies. Here, we describe an *in vitro* cell culture system, using CD40-CD154 interactions, that leads to a 1×10^6^-fold expansion of switched memory B lymphocytes in approximately 50 days. These expanded cells secrete polyclonal IgG, which distribution into IgG_1_, IgG_2_, IgG_3_ and IgG_4_ is similar to that of normal human serum. Such *in vitro* generated IgG showed relatively low self-reactivity since they interacted moderately with only 24 human antigens among a total of 9484 targets. Furthermore, up to one liter of IgG secreting cells can be produced in about 40 days. This experimental model, providing large-scale expansion of human B lymphocytes, represents a critical step toward the *in vitro* production of polyclonal human IgG and a new method for the *ex vivo* expansion of B cells for therapeutic purposes.

## Introduction

Therapeutic immunoglobulins, or intravenous immunoglobulins (IVIg), are prepared from pools of plasma collected from more than 10 000 blood donors and are mainly constituted of IgG (≥98%) [1]. For more than 40 years, IVIg have been used as a replacement therapy in primary and secondary immune deficiencies [2]. Therapeutic immunoglobulins are also increasingly used in the treatment of autoimmune and inflammatory diseases in which they have been shown to re-establish the immune system’s homeostasis [3]. Their use in the treatment of inflammatory and autoimmune diseases and neurological diseases is continuously increasing, which would lead toa supplemental pressure on their supply [4]. IVIg consist of a large repertoire of polyclonal human IgG showing reactivity to pathogens as well as to human self-proteins [5]. Extensive investigations aiming at identifying specific IVIg immunomodulatory properties in order to eventually create substitutes to treat autoimmune and inflammatory diseases are currently being performed by several groups. Currently, a unique preparation of 25 monoclonal anti-RhD antibodies [6] is in phase II of clinical trials for the treatment of immune thrombocytopenic purpura (ITP) [7]. Further success in those clinical trials could qualify this monoclonal mix as a substitute for IVIg in ITP-treatment. However, polyclonal preparations for clinical applications are still the exception.

Essentially, patient’s accessibility to IVIg depends exclusively upon volunteer blood donation and there are no *in vitro* procedures allowing the preparation for these polyclonal human antibodies. Therefore, the development of an *in vitro* method for the production of large quantities of human IgG that could substitute for IVIg is highly relevant. As introduced above, *in vitro* generated human therapeutic antibodies are monoclonal and are mostly generated from transgenic mouse or genetic engineering such as chimeric, humanized or recombinant antibodies [8], [9], [10], [11]. Nevertheless, long-term cultures of human B lymphocytes have been proposed 20 years ago by Banchereau and collaborators while designing the CD40-CD154 culture system [12]. This co-culture model is based upon interactions between CD40 present on all B lymphocytes and CD154^+^ adherent cell line. The model was expected to allow the generation and clonal expansion of human B cell lines [13]. Since then, many groups have used this culture system to activate human B lymphocytes to study their physiological characteristics in relation to the immune response (reviewed in [14]). However, the concept of large expansion of B lymphocytes was not developed nor relevant until recently, when antigen-presenting capacity of B lymphocytes were viewed as an asset for cancer treatment [15], [16], [17].

Here, we report a model based upon CD40-CD154 interactions, enabling high levels of expansion as well as differentiation of human switched memory B lymphocytes. This long-term culture model could be a critical step toward a large-scale production of human IgG as well as *ex vivo* expansion of human memory B lymphocytes.

## Materials and Methods

### Preparation of Human Mononuclear Cells

This study has been approved by Héma-Québec’s Research Ethics Committee and every regular platelet donors who agreed to participate in this study, have signed an informed consent. Leukoreduction system (LRS) chambers from Trima Accel™ collection systems (Gambro BCT, Lakewood, CO, USA) were collected after routine apheresis. Leukocytes were recovered from LRS chambers, as previously described [18], and used to isolate peripheral blood mononuclear cells (PBMNCs) by centrifugation on Ficoll-Paque following manufacturer’s instructions (GE Healthcare, Baie d’Urfé, QC, Canada). PBMNCs were stored, frozen, until B lymphocytes preparation [18].

### Isolation of Peripheral B Lymphocytes

CD19^+^ B lymphocytes were isolated from PBMNCs by negative selection using StemSep™ or EasySep™ CD19 cocktail following manufacturer’s instructions (Stem Cell Technologies, Vancouver, BC, Canada). CD19^+^ B lymphocytes’ purity, as determined by flow cytometry, was higher than 95% in all experiments reported herein. Switched-memory B cells, namely IgG^+^ or IgA^+^ cells, were further isolated using an EasySep™ custom cocktail containing antibodies directed against IgD and IgM (Stem Cell Technologies). This two-step selection provided untouched B lymphocytes. IgD^+^IgM^+^ and IgM^+^ cells depletion was higher than 95% in all the assays.

### Human B-lymphocytes Culture

Switched-memory B cells were seeded at 3 to 4×10^5^ cells/mL in 6-well Primaria plates (BD Biosciences, Mississauga, Canada) in the presence of 0.5×10^5^ cells/cm^2^ γ-irradiated CD154^+^ L4.5 cells [19], [20]. The cells were cultured in IMDM supplemented with 10% ultra low IgG FBS containing 10 µg/mL insulin, 5.5 µg/mL transferrin, 6.7 ng/mL sodium selenite, 100 µg/ml streptomycin and 100 U/ml penicillin G (all from Invitrogen, Burlington, ON, Canada). The culture medium was supplemented with a mix of cytokines, namely 5 ng/mL IL-2 (∼50 U/mL), 40 ng/mL IL-10 (∼20 U/mL) (both from PeproTech, Rocky Hill, NJ, USA) and 100 U/mL IL-4 (R&D Systems, Minneapolis, MN, USA) which sustained expansion and differentiation of human switched-memory B cells [21], [22]. Cultures were fed by replacing at least half of the culture medium every 2–3 days. Gamma-irradiated adherent L4.5 cells were renewed every 4–5 days to maintain a constant ratio of about 5 B cells per CD154^+^ L4.5 cell, which corresponds to about 500 to 2000 CD154 molecules per B cell [23]. Cell counts and viability were evaluated in triplicates by Trypan blue exclusion using a hemocytometer. Expansion factor (EF) was calculated, between time 2 (t_2_) and time 1 (t_1)_, by using the cellular density (D) and total number of seeded cells (N_1_) according to the following formula: E = [D_2_–D_1_]×N_1_. Generation time (t_gen_) was calculated within the initiation phase of the growth curve according to the formula: κ = 1/ln2 (ln[N_t2_] - ln[N_t1_])/t_2_-t_1_ and t_gen_ = 1/κ. EBV DNA was monitored in expanded B cells at the end of the culture period by RT-PCR amplification of *EBNA1,* as previously published [16].

### Mini-scale Cultures

When indicated, switched-memory B cell cultures were initiated as described above in 24- or 6-well plates in the presence of γ-irradiated L4.5 cells up to a total number of 4 to 5×10^6^ cells for their subsequent transfer into a Petri dish (100×20 mm, BD Biosciences). Cells were seeded at a final concentration of 3×10^5^ cells/mL, in a final volume of 15 mL per Petri dish, and expanded to obtain a final volume of approximately 0.5 L.

### Flow Cytometry Analyses

APC-conjugated anti-CD14, anti-CD19, anti-CD38 and anti-IgG, FITC-conjugated anti-CD3, anti-IgM and anti-IgA, PE-conjugated anti-CD45, anti-CD138 and anti-IgD, and PerCP-Cy5.5-conjugated anti-CD3 and anti-CD19, were IgG_1_ mouse monoclonal antibodies obtained from BD Biosciences. FITC-conjugated anti-IgM (Jackson ImmunoResearch Laboratories, West Grove, PA, USA) and anti-IgA (AbD Serotec, Raleigh, NC, USA) were polyclonal goat antibodies. Cells were stained and fixed with 2% paraformaldehyde (Sigma-Aldrich, Oakville, ON, Canada). All analyses were done on 5000 to 10000 viable cells gated in a region determined by 7-amino-actinomycin-D (7AAD, BD Biosciences). Analyses were performed using a FACSCalibur flow cytometer and the CellQuest software (BD Biosciences). Data were subsequently analyzed using FCS Express II (De Novo Software, Los Angeles, CA, USA).

### Determination of Immunoglobulin Concentrations

IgA, IgG, IgG_1_, IgG_2_, IgG_3_ IgG_4_ and IgM concentrations in culture supernatants were determined by ELISA. Goat affinity-purified antibodies specific to human Fc fragment of IgA, IgG, IgM (Jackson ImmunoResearch Laboratories), IgG_1_ and IgG_3_ (Invitrogen), IgG_2_ (BD Biosciences) and IgG4 (Southern Biotech, Birmingham, USA) were used to capture the secreted immunoglobulins. IgG and IgM were revealed using peroxidase-conjugated goat antibodies against human Ig (Jackson ImmunoResearch Laboratories). IgA were revealed with peroxidase-conjugated goat anti-human antibodies specific to the alpha chain while IgG subclasses were revealed using goat anti-human antibodies specific for the gamma chain of the Fc fragment. IgE concentrations were determined using Human IgE Ready-SET-Go following manufacturer’s instructions (eBioscience Inc., San Diego, USA), Secretion rates were determined using washed cells seeded at 2×10^6^cells/ml for 20 to 22 hours without cytokines or L4.5 cells. This culture’s supernatant was then used in an ELISA assay as described above.

### Immunoglobulins Analysis by Isoelectrofocusing

Thin-layer isoelectrofocusing (IEF) was performed on a 5% acrylamide gel containing Bio-Lyte 3/10 ampholytes (Bio-Rad Laboratories, Mississauga, ON, Canada). Culture supernatants were compared to an in-house human monoclonal IgG and to a commercial intravenous immunoglobulin preparation (IVIg) (GamunexTM, Talecris Biotherapeutics ltd., Toronto, ON, Canada). A total of 100 ng IgG per samples and controls, according to IgG concentration determined by ELISA, were focused in three steps, consisting of 100V for 15 minutes, 200V for 15 minutes and 450V for one hour. Protein standards with pI ranging from 4.45 to 9.6 were used to monitor the focusing process (Bio-Rad Laboratories, Mississauga, Canada). According to a standard western blot assay, proteins were transferred from gels to Amersham Hybond-ECL nitrocellulose (GE Healthcare, Piscataway, USA) and membranes were revealed using peroxidase-conjugated goat antibodies specific to human γ chains (Jackson ImmunoResearch Laboratories). Detection was done with the Amersham ECL™ Western blotting detection reagents chemiluminescence kit (GE Healthcare), following the manufacturer’s instructions.

### Human Protein Microarray

The reactivity of the *in vitro* generated human IgG was determined by antibody specificity profiling service of Invitrogen (Carlsbad, USA). A pool of human IgG, prepared from the cumulated supernatants of 13 independent long-term cultures, was probed by Invitrogen at 0.1 µg/ml and 1.0 µg/mL. The ProtoArray Human Protein Micorarrays v5.0 was used to investigate 9484 human proteins. Data analysis was done by Invitrogen using ProtoArray Prospector software. The average background signal was 103 and 104 RFU (Relative fluorescence unit) for IgG samples adjusted to 0.1 and 1.0 µg/mL. Significant interactions between IgG and each targeted protein was based on three criteria established by Invitrogen. First of all, the test signal value was greater than 1000 RFU and more than 2-fold higher than the negative signal control for each protein. Second, the negative control signal value was less than 1000 RFU and finally, the replicate spot coefficient of variation (CV) was less than 50%.

## Results

### Large Expansion of Switched Memory B Lymphocytes

**Figure 1:**
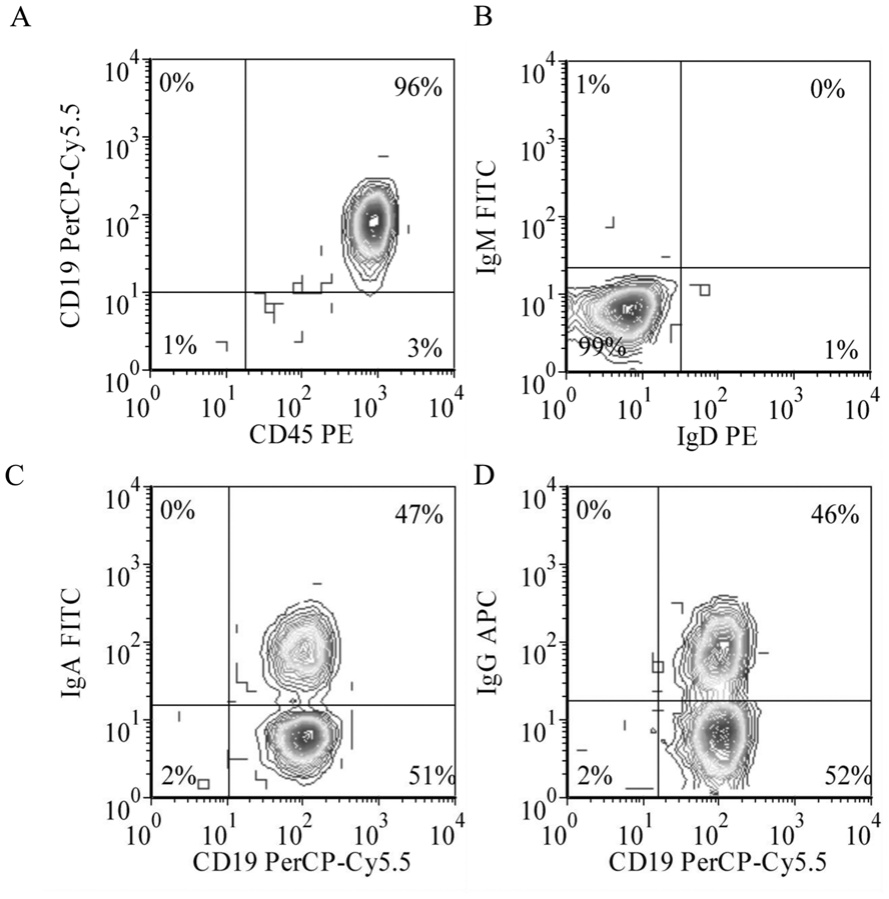
Selection of switched-memory B lymphocytes. In all experiments, purified CD19^+^ B lymphocytes (A) were depleted for IgD^+^IgM^+^ and IgM+ cells in a two-step selection process (B). Analysis of the resulting cell populations showed a relatively similar proportion of cells with surface IgA (C) and IgG (D).

Blood CD19^+^ B lymphocytes were depleted for IgD^+^ and IgM^+^ cells, giving a switched memory B lymphocyte population comprised of IgG^+^ and IgA^+^ cells, 54% ±11% and 36% ±12% respectively (Fig. 1), namely the IgG/IgA B lymphocyte population. The ratio of IgG and IgA was approximately 1.5, which is close to what is observed for small B cells in human blood [24], possibly resulting from the elutriation process used to remove mononuclear cells during platelet collection [18]. For all 13 samples presented in this study, the residual frequency of IgD^+^ and/or IgM^+^ cells was always less than 3% at the initiation of the culture. During the culture period, residual IgD^+^ cells remained lower than 3%, however, the frequency of IgM^+^ cells reached 10% ±4% in some experiments.

**Figure 2:**
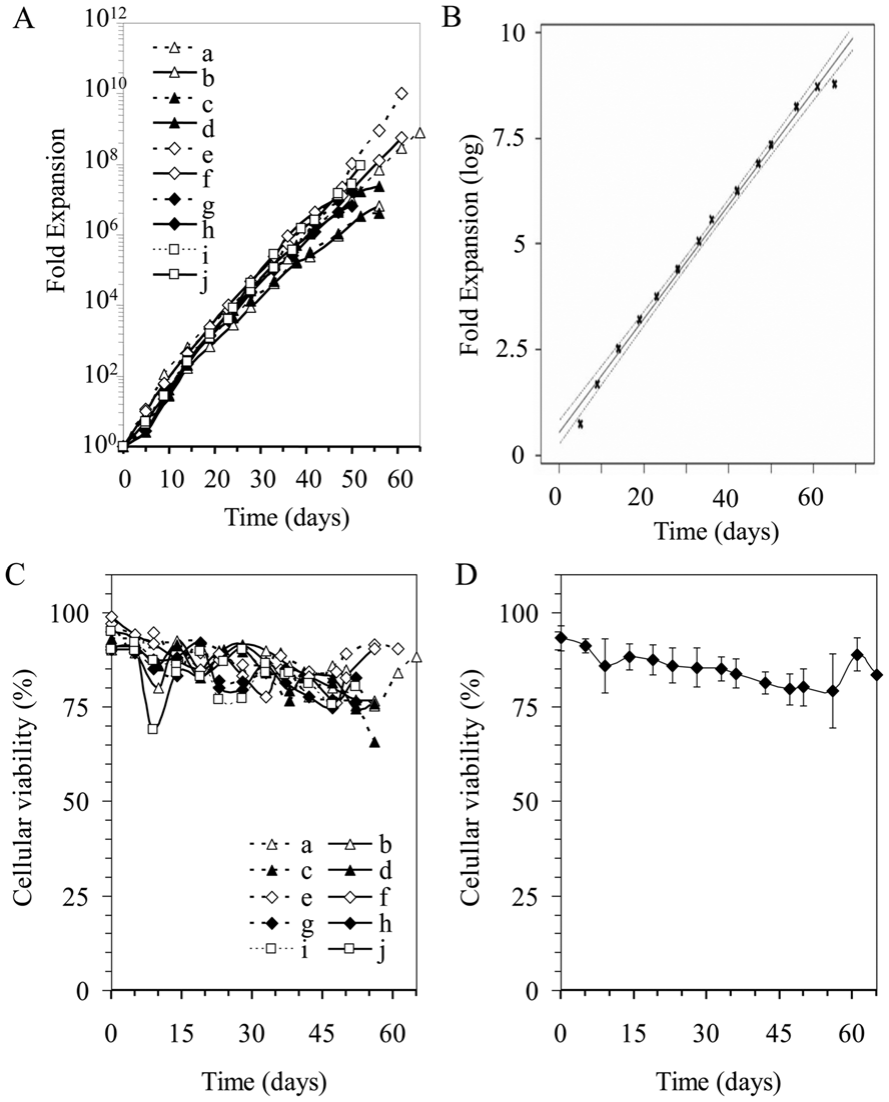
Long-term expansion of switched-memory B lymphocytes. (**A**) Ten samples of switched-memory B lymphocytes were cultured for 35 to 65 days in the presence of IL-2, IL-4, IL-10 and CD154^+^ cells (L4.5 cells) at a ratio of five B cells per L4.5 cell. Expansion factors for the ten independent samples were plotted as a function of time (days) in culture. (**B**) Regression analysis of the ten exponential growth curves presented in (A) resulted in a correlation coefficient of 0.9965 corresponding to the equation y = 10 (0.1344x+0.5415). (**C**) The proportion of viable cells during long-term culture was monitored on a regular basis for each culture. (**D**) The mean value and standard deviation of viability (%) showed a similar evolution for each samples.

Ten independent IgG/IgA B lymphocyte samples were isolated and stimulated in the presence of high levels of CD154 interaction and a mix of IL-2, IL-4 and IL-10 for 36 to 65 days. In order to evaluate the total expansion factor (Fig. 2A) as well as viability (Fig. 2C and D), IgG/IgA B lymphocytes were maintained in the exponential growth phase and their number was determined at the indicated days. The regression analysis of these ten exponential growth curves (Fig. 2B) showed a 0.9965 coefficient, indicating that the expansion was similar in relation to time and consistent among these experiments. The T_gen_ period calculated between days 28 to 36 in all these cultures, corresponded to a mean of 51 h±9 h (data not shown). Cell viability was also comparable from one cultured sample to another and maintained at an acceptable level, ranging from 93% to 80% at the end of the culture period (Figs. 2C and D). Overall, these culture conditions allowed a final expansion factor, based on the expansion rate and seeding cell numbers, ranging from 10^7^ to 10^9^ after 50 to 65 days.

### Expanded Switched-memory B Lymphocytes Contained Functional Ig-secreting Cells

**Figure 3:**
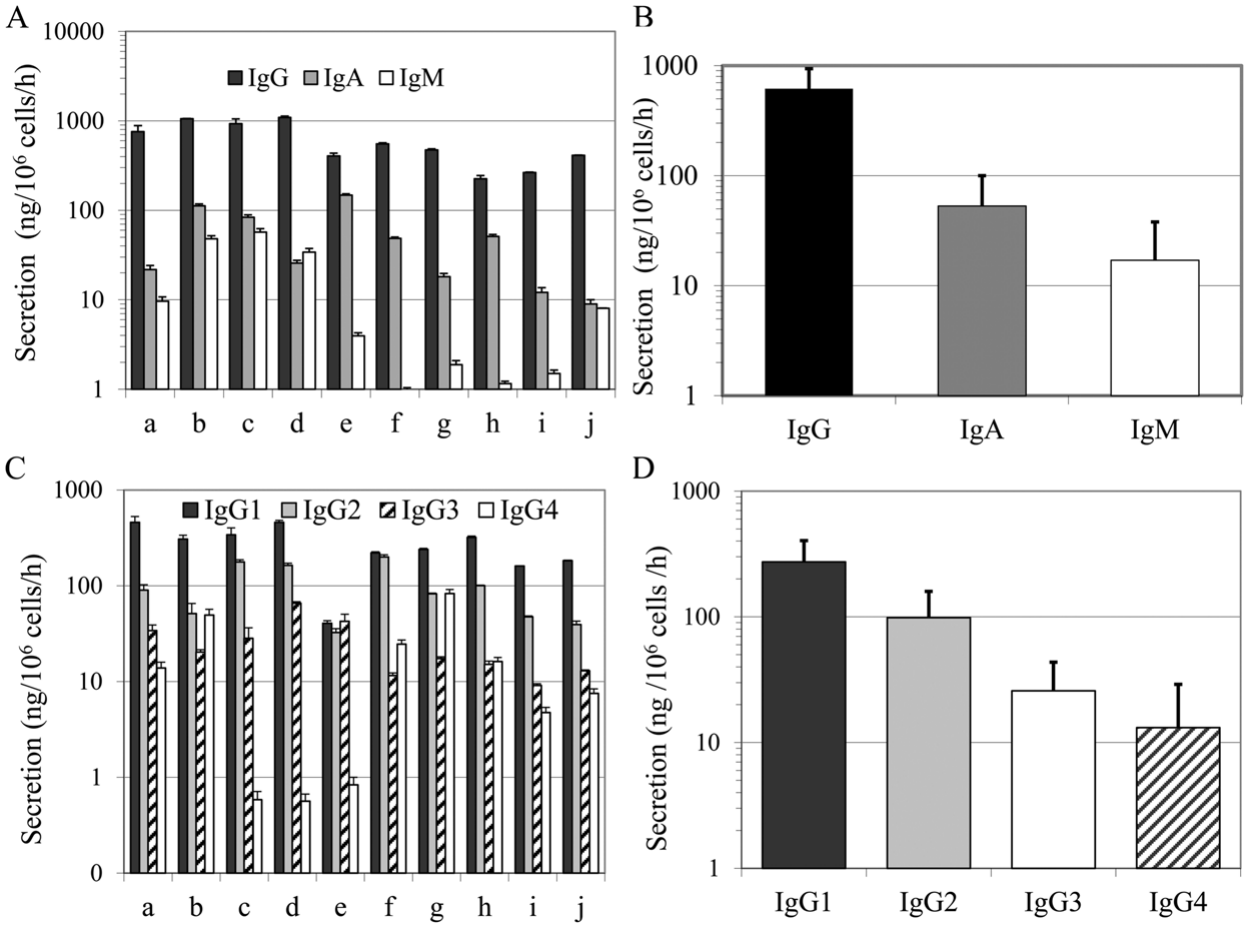
Switched-memory B lymphocytes secrete mostly IgG. Cells from the same ten independent experiments shown in Fig. 2, were collected during the exponential phase, namely on days 28 (e, f), 33 (b, c, d) 35 (a), and 37 (g, h, i, j), and seeded in fresh IMDM at 1–2×10^6^ cells/mL for 20–22 h. For each experiment, secretion rates were determined for IgA, IgG and IgM (A, B) and IgG_1_, IgG_2_, IgG_3_ and IgG_4_ (C, D). Data are presented as the mean ± SD.

In order to estimate the differentiation status of the expanded IgG/IgA B lymphocytes, the secretion rates for IgG and IgA were determined during the exponential phase, i.e. between day 28 and day 37. IgM secretion was also measured in the supernatant as a supplemental control for negative selection efficiency and to verify whether the frequency of IgM^+^ B lymphocytes increased (Fig. 3A and B). As expected, all ten experiments showed very low levels of IgM secretion, ranging from 1 to 57 ng/10^6^cells/h, which corresponds to less than 5% (mean: 2% ±2%) of total Ig secretion. Therefore, more than 95% of secretion was related to switched-memory B lymphocytes. Except for samples e and h, which had IgA secretion rates of 25–30% of total secretion, IgG secretion was predominant with more than 87% of all Ig secreted (mean 90% ±8%). IgG’s secretion rates ranged from 200 to 1000 ng/10^6^ cells/h (Fig. 3A). The mean secretion rates for IgG, IgA and IgM were 618±321 ng/10^6^ cells/h, 53±47 ng/10^6^ cells/h and 17±21 ng/10^6^ cells/h, respectively (Fig. 3B), showing a very similar evolution in the differentiation status for all the tested samples.

### Secreted IgG Contains All Four Gamma Isotypes

The secretion rates for the gamma isotypes were determined using specific ELISA tests (Fig. 3 C and D). The secretion rates varied from 160 to 460 ng/10^6^cells/h for IgG_1_, from 39 to 199 ng/10^6^cells/h for IgG_2_, from 9 to 66 ng/10^6^cells/h for IgG_3_ and from 1 to 83 ng/10^6^cells/h for IgG_4_ (Fig. 3 C). According to the secretion rate mean values for the ten experiments (Fig. 3D), the relative proportions of IgG_1_ (67%), IgG_2_ (24%), IgG_3_ (6%) and IgG_4_ (3%) were comparable to those reported in human serum, namely IgG_1_ 60% ±15%, IgG_2_ 30% ±5%, IgG_3_ 7% ±2% and IgG_4_ 3% ±1% [25], [26]_._ The IgG total secretion recorded for each culture experiments ranged from 30 to 115 µg/mL (data not shown). Overall these data showed that long-term culture of IgG^+^ human B lymphocytes did not induce a bias in the secretion of IgG isotypes, which was consistent with the reported proportions in human blood.

### Expanded IgG^+^ B Lymphocytes are Polyclonal Populations

**Figure 4:**
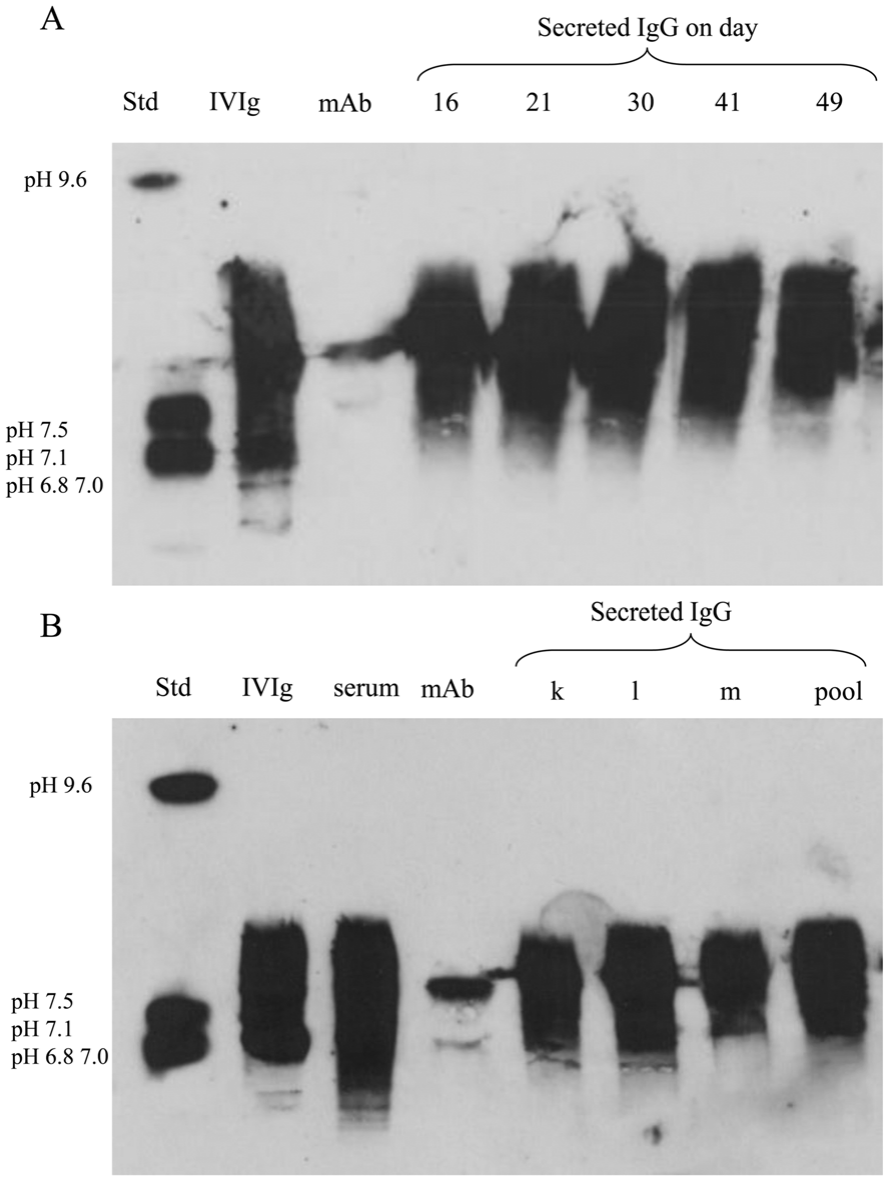
Switched-memory B lymphocytes secrete polyclonal IgG. IgG’s polyclonality was determined by IEF for experiments a to j. A representative pattern is presented in panel A. (A) Briefly, IEF standard (Std), IVIg, a human monoclonal IgG (mAb) and culture supernatants from experiment a sampled on days 16, 21, 30, 41 and 49 are shown. This polyclonal IEF pattern is similar for the ten independent cultures presented in Fig. 1. (B) IEF pattern was determined for the cumulated supernatants of the three experiments presented in Fig. 5 as well as the pooled supernatants of 13 representative experiments. As mentioned above, Std, IVIg and mAb were used as controls and a sample from a healthy human serum was also used for the same purpose. For all samples in (A) and (B), analyses were done on 100 ng IgG per well.

The degree of heterogeneity of secreted IgG molecules was assessed during the long-term culture by sampling culture supernatant at various time points and analyzing their patterns by isoelectrofocusing. Analysis of secreted IgG from day 16 to day 49 (fig. 4A) showed smears of IgG bands, which are characteristic of polyclonal IgG and similar to the IgG IEF pattern of IVIg. All ten experiments (a to j), showed similar patterns of polyclonality indicating that the expanded IgG^+^ B-lymphocyte population maintained its diversity, even after long-term culture. The presence of *EBNA1* was determined only on samples *e* to *j* (Fig. 2) and 3 of them (e, f, h) were found positive (data not shown).

### Validation of Expansion of Switched-memory B Lymphocytes

**Figure 5:**
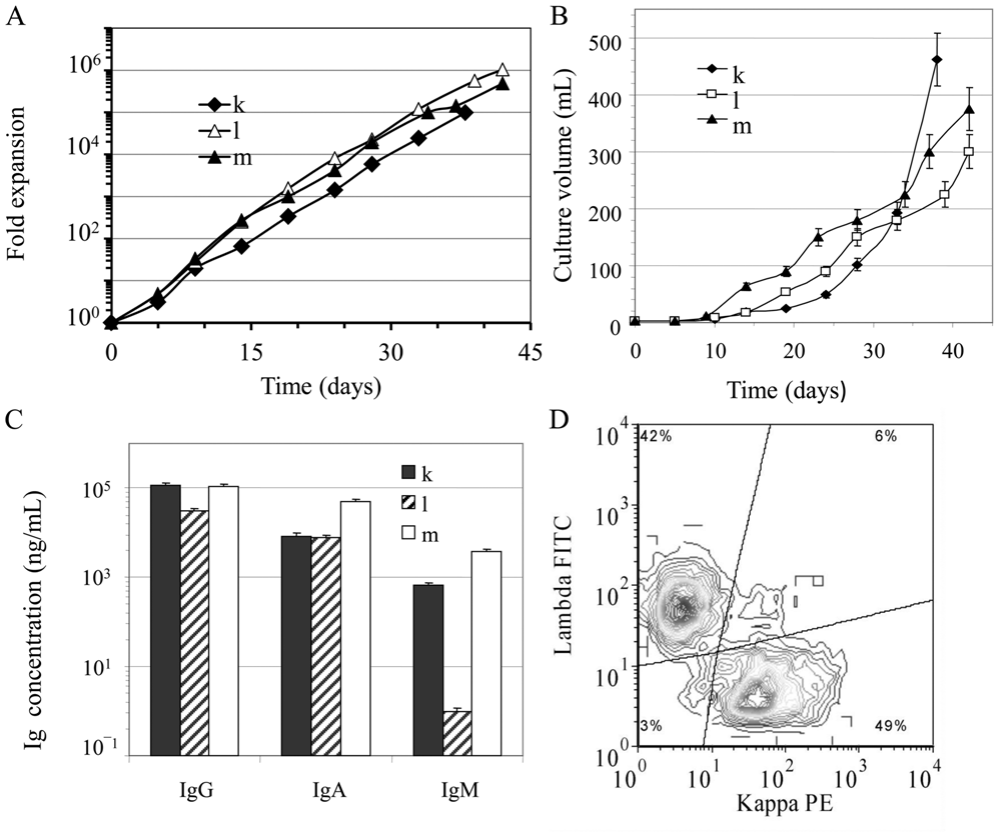
Validation of expansion during long-term culture. Three switched-memory B lymphocyte samples were cultured as described in Fig. 1 and transferred in petri dishes to test the feasibility of increasing the culture volume up to 500 mL. (**A**) Expansion factors were similar to those obtained in 6-well plates (Fig. 1). (**B**) Culture volumes are shown as a function of time. (C) IgA, IgG and IgM concentrations were determined in supernatants of the three independent samples at the end of the culture. (D) Flow cytometry analyses for kappa and lambda chain expression was similar for all three independent samples.

The ability of switched-memory B lymphocytes to expand in larger culture volumes was assessed by serial passaging of three long-term cultures from 6-wells plates to petri dishes. A culture period of 35 to 40 days could be easily achieved, and allowed to reach real culture volumes up to 300 to 450 ml. Cellular densities were maintained between 4×10^5^ cells/ml and 3×10^6^ cells/ml, which added up to more than 10^9^ total switched-memory B lymphocytes at the end of the culture experiment (Fig. 5 A and B). The three independent samples presented in Figure 5 (k, l and m) expanded in larger volumes, showed expansion rates similar to those observed above (Fig. 1) and to experiments done with the same samples cultured in 6-well plates (data not shown). The presence of *EBNA1* was detected in expanded cells originated from sample *l*, whereas those generated with samples *k* and *m* were found negative (data not shown).

In these experiments, the cumulated supernatants were separately pooled and the total secretion of IgG, IgA, and IgM ranged from 30 to 116 µg/mL, 8 to 49 µg/mL and 1 to 3700 ng/mL, respectively (Fig. 5C). As above, IgM concentration represented less than 4% while IgG consisted of 67% to 93% of total Ig secretion. IEF analysis of these cumulated supernatants, separately and as a pool, also showed a polyclonal IgG distribution (Fig. 4B), which was similar to that of IVIg as well as IgG present in human serum. Flow cytometry analysis of the expanded cells showed acceptable proportions of kappa and lambda light chain (Fig. 5D) [27] and, as above, all four gamma isotypes were present with frequencies declining from IgG_1_ to IgG_3_/IgG_4_ (data not shown). Lastly, the total IgG secretion of the pooled culture supernatants reached 30 mg to 100 mg of human IgG in final volumes of 0.7L to 1L.

### In vitro Generated IgG are Weakly Self-reactive

The protein microarray assay was used to determine whether or not the *in vitro* generated IgG showed self-reactivity [28]. A pool of culture supernatants from 13 independent experiments, which displayed a polyclonal pattern (Fig. 4B) and which was constituted of 75% IgG, was probed on 9484 human peptides. A total of 24 targets were spotted by the IgG preparation (Table 1), which represents 0.2% of the 9484 peptides tested. As a comparison, the screening of a commercial IVIg using the same protein array assay reacted with 67 targets [29] including 10 of those listed in Table 1 as indicated (#). Besides, when using switched-memory B lymphocytes, obtained from participants recently vaccinated for hepatitis B, we detected IgG specific reactivity for hepatitis B surface antigen in our culture supernatants (data not shown).

**Table 1:** Human polyclonal IgG interacting proteins using a protein array.

Accession No.	Signal^1^IgG		CV^1^(%)	IgG/neg^1^	Description^2^
NM_002903.1		7064	2	345	recoverin (RCVRN) (*)
NM_004987.3		6499	4	24	LIM and senescent cell antigen-like-containing domain protein 1 (*) (#)
NM_133491.2		4253	1	41	spermidine/spermine N1-acetyltransferase 2 (SAT2
BC026346.1		3891	2	71	family with sequence similarity 84, member A (FAM84A) (*)
PV3850		3499	4	8	casein kinase 1, alpha 1 (CSNK1A1), transcript variant 1
PV3836		2887	3	1155	inhibitor of kappa light polypeptide gene enhancer in B-cells, kinase beta (IKBKB)
BC017865.1		2714	3	10	Fc fragment of IgG, low affinity IIIa, receptor (CD16a) (FCGR3A) (*) (#)
NM_018184.1		2707	6	7	ADP-ribosylation factor-like 8B (ARL8B) (#)
BC020229.1		2442	17	71	arylsulfatase D (ARSD) (#)
NM_014288.2		1854	3	3	centromere protein R (#)
NM_017614.3		1807	16	2157	betaine-homocysteine methyltransferase 2 (BHMT2)
NM_002150.1		1687	4	427	4-hydroxyphenylpyruvate dioxygenase
BC036723.1		1535	12	74	Fc fragment of IgG, low affinity IIIa, receptor (CD16a) (FCGR3A) (#)
BC016768.1		1491	0	507	nucleophosmin (nucleolar phosphoprotein B23, numatrin) (NPM1)
PV3144		1487	6	450	neurotrophic tyrosine kinase, receptor, type 1 (NTRK1), transcript variant 3
NM_007030.1		1259	3	24	tubulin polymerization promoting protein (TPPP)
BC018929.1		1197	8	2	pleckstrin homology-like domain, family A, member 1 (PHLDA1) (#)
NM_018246.1		1188	6	5	coiled-coil domain containing 25 (CCDC25)
NM_001007246.1		1160	1	4	bromodomain and WD repeat domain containing 1 (BRWD1), transcript variant 3
NM_138565.1		1109	2	2	cortactin (CTTN), transcript variant 2 (#)
NM_138809.1		1057	4	8	carboxymethylenebutenolidase homolog
NM_004450.1		1041	1	5	enhancer of rudimentary homolog (Drosophila) (ERH)
NM_152328.3		1018	2	2	adenylosuccinate synthase like 1 (ADSSL1), transcript variant 2 (#)
NM_133265.2		1015	7	3	angiomotin (AMOT) (#)

1 Data present the IgG signal (1.0 µg/mL; threshold >1000) corresponding to background subtracted pixel intensity value (RFU), the CV associated with the duplicate spots for each protein and the fold increase between IgG and negative control signals.

2 (*) Indicated significant interaction when IgG was adjusted to 0.1 µg/ml concentration (Threshold >500 RFU).

3 (#) Indicated significant interaction with a commercial intravenous immunoglobulin preparation (IVIg) adjusted to 1.0 µg/mL (Threshold >1000 RFU).

### In vitro Expanded Switched-memory B Lymphocytes Generated IgE-secreting Cells

IgE^+^ B lymphocytes are expected to be of very low frequency in peripheral blood B lymphocytes; however we found out that the mean concentration of IgE in the above pool of 13 supernatants was 12.5±2.2 µg/mL. We also tested cumulated supernatants from 3 independents experiments and obtained similar IgE concentrations, namely 13.3±2.2 µg/mL, 5.0±0.2 µg/mL and 7.4±0.5 µg/mL.

## Discussion

The present study established long-term culture conditions enabling the generation of large quantities of human B lymphocytes. The resulting pool of human B lymphocytes, which could be enlarged by up to 10^6^-fold after 45 to 50 days, were polyclonal and viability was still very good (>80%). These populations included proportions of gamma isotypes as well as kappa/lambda ratios that were comparable to those observed in human blood [26], [27], [30]. Overall, this *in vitro* culture model allows the generation of large amounts of B lymphocytes as well as their utilization for the production of IgG and/or IgA.

The polyclonal progression of B lymphocytes in these 13 experiments is crucial since it opens to the possibility to have access to a large human antibody repertoire. Banchereau’s group was the first to report the culture of human B lymphocytes for as long as 10 weeks [13]. Thereafter, several groups have used CD40-activation to perform long-term expansion of unsorted blood B lymphocytes for cellular immunotherapy [16], [31], [32], [33]. Among them, Wiesner’s group has done exhaustive investigations of the resulting B lymphocyte populations. Overall, their strategy provided a B lymphocyte expansion ranging from 100- to 1000-fold after 40 days that could be maintained for up to 400 days. However, although most cultured cells were EBV-negative, their analysis of kappa/lambda ratios revealed an oligoclonal expansion of human B lymphocytes, suggesting the domination of some subsets [16]. We already showed that upon CD40-activation, naïve B lymphocytes were prone to dominate the culture [34] and were able to inhibit memory B lymphocyte expansion [35]. In the present study, by using purified switched-memory B lymphocytes, we eliminated such negative modulation and allowed the switched-memory to expand rapidly following high levels of CD40-CD154 interactions for up to 2 months. Besides, we observed that IgA secretion was rapidly decreasing during the three weeks of culture (data not shown). In fact, in all our cultures, IgG was dominant representing 70% to 90% of all secreted immunoglobulins suggesting that proliferation and differentiation of IgG^+^ cells were steadier than that of IgA^+^ cells in our long-term culture conditions. However, we also observed that the proportion of IgE secretion, which may represent about 2% of the purified switched-memory B lymphocytes in our cultures, can be close to that of IgA indicating that these culture conditions were favorable for IgE^+^ B lymphocytes.

Conversely, the possibility that EBV^+^ human B lymphocyte clones could emerge from long-term cultures might generate a bias in the B lymphocyte repertoire [13], [16], [36]. In this study, 4 out of 9 expanded switched-memory B cells were positive for *EBNA1* at the end of the culture period. This was expected since the virus persists in the memory B lymphocyte compartment [37], [38], [39]. Although 95% of Caucasian adults are healthy virus carriers, EBV^+^ cells are rare events, ranging from 1 to 50 positive cells per 1×10^6^ blood B lymphocytes [40], [41]. Recently, EBV^+^ B lymphocytes undergoing germinal center reaction in human tonsils were shown to depend on a balance between proliferation and cell death, resulting in a stable number of infected cells [41]. The long-term cultures described here used the CD40-CD154 interaction, which is a central player in the germinal center reaction [12] and thus might result in a similar persistence of EBV^+^ cells without enlarged frequency. Besides, the fact that our cultures did not show oligoclonal but polyclonal patterns, suggests that the EBV^+^ B lymphocytes were not advantaged during the long-term expansion.

The concept of human polyclonal antibodies is still a perspective for the future development of therapeutic antibodies [8]. A few years ago, transgenic animals were proposed as factories to replace the immunized polyclonal antibodies prepared from human or animal plasma [42]. Polyclonal human antibodies have indeed been produced in transgenic cow and were reported to efficiently inactivate bacterial toxins [43]. Nevertheless, a mixture of monoclonal antibodies is actually the most promising product for therapeutic use in human [6], [7], [8].

In this study, we worked with frozen mononuclear cells to facilitate the technological transfer to an industrial process. However such long-term cultures can be done with freshly isolated PBMNCs from the beginning to the final step. Whether or not the human IgG^+^ repertoire may be influenced by a freezing step remains to be determined, but we already have good indications that a large sampling of this repertoire is present in the cultured cells.

In conclusion, the culture system described here allows the expansion of 100,000 selected B lymphocytes to a total of 10^8^ cells in less than 3 weeks as well as the production of milligrams of polyclonal antibodies in less than 1000 mL. This system could be considered as a new way to exploit the human repertoire. Furthermore, these culture conditions might be converted for the utilization of hollow fibre bioreactors to prepare larger quantities of human polyclonal IgG. These expanded cells could also become a new source of human memory B lymphocytes, as the enlarged quantities produced could be an asset in single cell antibody technologies [44] to prepare a mixture of monoclonal human antibodies. Finally, these culture conditions could allow the expansion of autologous effector human B lymphocytes to be used in cell-based therapies.
